# Supplementary material for: The asymmetry of female meiosis reduces the frequency of inheritance of unpaired chromosomes
Source: eLife. 2015 Apr 7;4:e06056. doi: 10.7554/eLife.06056 (PMC4412107; doi:10.7554/eLife.06056)
Supplement: Supplementary file 1. — C. elegans strains used in this study. DOI: http://dx.doi.org/10.7554/eLife.06056.014 [file elife-06056-supp1.docx]

**Supplementary File 1: *C. elegans* strains used in this study**

| **Strain Name** | ***Genotype*** | **Source** | **Progeny Counts (XO, XX, XXX, Dead)** |
| --- | --- | --- | --- |
| CB1489 | *him-8 (e1489) IV* | Hodgkin *et al.* 1979 | 36.7%, 56.1%, 6.4%, 0.8% (Hodgkin *et al*.; Table 2) |
| FM125 | *unc-119 (ed3); ruls57 [pAZ147: pie-1/B-tubulin::GFP; unc-119 (+)]; itls37 [unc-119(+) pie-1::mCherry::H2B]* | Ellefson and McNally, 2011 | 0.03%, 99.04%, 0%, 0.93% |
| FM126 | *unc-119 (ed3); ruls57 [pAZ147: pie-1/B-tubulin::GFP; unc-119 (+)]; itls37 [unc-119(+) pie-1::mCherry::H2B]; him-8 (e1489) IV* | Crossed FM125 with CB1489 | 36.88%, 55.57%, 3.44%, 4.11% |
| OD27 | *l* *ltIs14[pASM05: pie-1::GFP-TEV-STag::air-2 + unc-119(+)]* | Lewellyn et al. 2011 |  |
| FM197 | *ltIs14[pASM05: pie-1::GFP-TEV-STag::air-2 + unc-19(+)]); him-8 (e1489) IV* | Crossed OD27 with CB1489 |  |
| FM232 | *ltIs37 [pAA64; pie-1::mCherry::HIS-58 + unc-119(+)] him-8 (e1489) IV; ltIs38 [pAA1; pie-1::GFP::PH(PLC1delta1)+ unc-119(+)]* | Crossed FM126 with OD95 | 36.74%, 56.17%, 3.54%, 3.54% |
| OD95 | *ltIs38 [pAA1; pie-1::GFP::PH(PLC1delta1) + unc-119(+)]* | McNally et al., 2006 |  |
| TY5434 | *syTs44(LacO) V* | Severson and Meyer, 2014 |  |
| W354 | *zim-2 (tm574) IV; syTs44(LacO) V)* | crossed TY5434 with CA258 (*zim-2 (tm574)*)  (Checci et al., 2014) | 1.58%, 74.60%, 0%, 23.81% |
| EG7477 | *syIS46 [hsp16::gfp-LacI; dpy-30::S65TGFP; LacO; dpy-20(+)] II ; unc-119(ed3) III ; dpy-20(e1282ts) IV; oxTi483[LacO unc-119 (+) PuroR] X* | Frøkjær-Jensen et al., 2014 |  |
| FM299 | *unc-119(ed3)III; oxTi483[LacO unc-119 (+) PuroR] X* | Crossed EG7477 with N2 |  |
| FM300 | *unc-119(ed3)III; oxTi483[LacO unc-119 (+) PuroR] X; him-8(e1489)IV* | Crossed FM299 eith CB1489 | 40.23%, 53.62%, 3.76%, 2.39% |
| AV494 | *WT 3X:2A* | Mlynarczyk-Evans *et al.* 2013 | 0%, 58.26%, 36.65%, 5.08% |
| BW305 | *lon-2(e678) X* |  |  |
| HR604 | *mel-11(it26) unc-4(e140)/mnC1 II* |  |  |
| HR1605 | *mel-11(it26) unc-4(e140)/mnC1 II; him-8(e1489) IV* |  |  |
| FM13 | *mei-2 (ct98)* I; *ruls57 [pAZ147: pie-1/B-tubulin::GFP; unc-119 (+)]; itls37 [unc-119(+) pie-1::mCherry::H2B]; him-8 (e1489) IV* | McNally et al., 2006 |  |
| FM316 | *mei-2 (ct98)* I; *ruls57 [pAZ147: pie-1/B-tubulin::GFP; unc-119 (+)]; itls37 [unc-119(+) pie-1::mCherry::H2B]* | Crossed FM13 with CB1489 |  |
