# Supplementary figures and images for: The asymmetry of female meiosis reduces the frequency of inheritance of unpaired chromosomes
Source: eLife. 2015 Apr 7;4:e06056. doi: 10.7554/eLife.06056 (PMC4412107; doi:10.7554/eLife.06056)

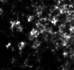

Supplement: Supplementary file 2. — Z-stack of XC FISH on XXX wild-type metaphase plate in meiosis I. 16-bit 3-channel TIFF can be opened using FIJI or basic ImageJ (http://fiji.sc/Downloads). Data shown are a z-stack acquired with 300 nm steps through a meiosis I metaphase spindle. Chromosomes are shown in blue (DAPI), tubulin antibodies label the spindle in green, and the XC FISH probe labels X chromosomes (2 present) in red. Channels can be split for individual analysis using the channel splitter (Image > Colors > Split channels) or can be hidden using the channels tool (Image > Colors > Channels tool). DOI: http://dx.doi.org/10.7554/eLife.06056.015 [file elife-06056-supp2.zip › elife-06056-supp2-v2-download (2).tif]

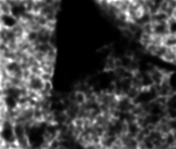

Supplement: Supplementary file 3. — Z-stack of XC FISH on XXX wild-type metaphase plate in meiosis II. 16-bit 3-channel TIFF can be opened using FIJI or basic ImageJ (http://fiji.sc/Downloads). Data shown are a z-stack acquired with 300 nm steps through a meiosis II metaphase spindle. Chromosomes and the first polar body, which is on the top, are shown in blue (DAPI), tubulin antibodies label the spindle in green, and the XC FISH probe labels X chromosomes (1 present on the spindle) in red. Channels can be split for individual analysis using the channel splitter (Image > Colors > Split channels) or can be hidden using the channels tool (Image > Colors > Channels tool). DOI: http://dx.doi.org/10.7554/eLife.06056.016 [file elife-06056-supp3.zip › elife-06056-supp3-v2-download.tif]
